# 3-[(2-Hydroxy­ethyl)imino­meth­yl]-1,1′-bi-2-naphthol

**DOI:** 10.1107/S1600536809016407

**Published:** 2009-05-07

**Authors:** Yu Zhang, Kun Wang, Ling-Zhi Zhong, Rui-Xiang Li

**Affiliations:** a Institute of Homogeneous Catalysis, Department of Chemistry, Sichuan University, Chengdu 610064, People’s Republic of China

## Abstract

In the title compound, C_23_H_19_NO_3_, there is an intra­molecular O—H⋯N hydrogen bond, which forms a six-membered ring, and inter­molecular O—H⋯O hydrogen bonds stabilize the crystal structure.

## Related literature

For background on the application of salen complexes to asymmetric catalysis, see: Pu (1998 ▶). For the synthesis of the title compound, see: Chin *et al.* (2004 ▶).
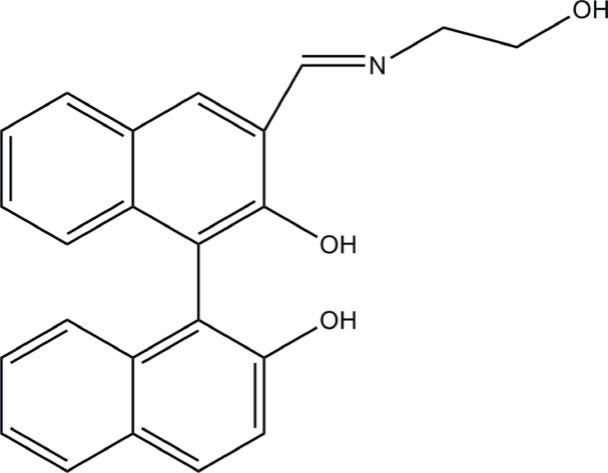


## Experimental

### 

#### Crystal data

C_23_H_19_NO_3_
*M* *_r_* = 357.39
Orthorhombic, 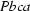

*a* = 12.6184 (3) Å
*b* = 9.7774 (2) Å
*c* = 29.7991 (6) Å
*V* = 3676.47 (14) Å^3^
*Z* = 8
Mo *K*α radiation
μ = 0.09 mm^−1^
*T* = 296 K
0.50 × 0.40 × 0.36 mm

#### Data collection

Bruker SMART CCD area-detector diffractometer
Absorption correction: multi-scan (*SADABS*; Sheldrick, 2004 ▶) *T*_min_ = 0.661, *T*_max_ = 1.000 (expected range = 0.641–0.970)
24940 measured reflections
4220 independent reflections
1912 reflections with *I* > 2σ(*I*)
*R*_int_ = 0.089

#### Refinement

*R*[*F* ^2^ > 2σ(*F* ^2^)] = 0.055
*wR*(*F* ^2^) = 0.135
*S* = 1.00
4220 reflections
252 parameters
H atoms treated by a mixture of independent and constrained refinement
Δρ_max_ = 0.15 e Å^−3^
Δρ_min_ = −0.14 e Å^−3^

### 

Data collection: *SMART* (Bruker, 1997 ▶); cell refinement: *SAINT* (Bruker, 1997 ▶); data reduction: *SAINT*; program(s) used to solve structure: *SHELXS97* (Sheldrick, 2008 ▶); program(s) used to refine structure: *SHELXL97* (Sheldrick, 2008 ▶); molecular graphics: *SHELXTL* (Sheldrick, 2008 ▶); software used to prepare material for publication: *SHELXTL*.

## Supplementary Material

Crystal structure: contains datablocks I, global. DOI: 10.1107/S1600536809016407/bt2938sup1.cif

Structure factors: contains datablocks I. DOI: 10.1107/S1600536809016407/bt2938Isup2.hkl

Additional supplementary materials:  crystallographic information; 3D view; checkCIF report

## Figures and Tables

**Table 1:** Hydrogen-bond geometry (Å, °)

*D*—H⋯*A*	*D*—H	H⋯*A*	*D*⋯*A*	*D*—H⋯*A*
O2—H2*A*⋯O3^i^	0.82	1.87	2.6638 (17)	161
O3—H3*A*⋯O1^ii^	0.82	2.05	2.7724 (17)	147
O1—H1⋯N1	0.96 (2)	1.67 (2)	2.5649 (18)	153 (2)

Symmetry codes: (i) 
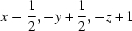
; (ii) 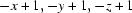.

**Fig. 1.**
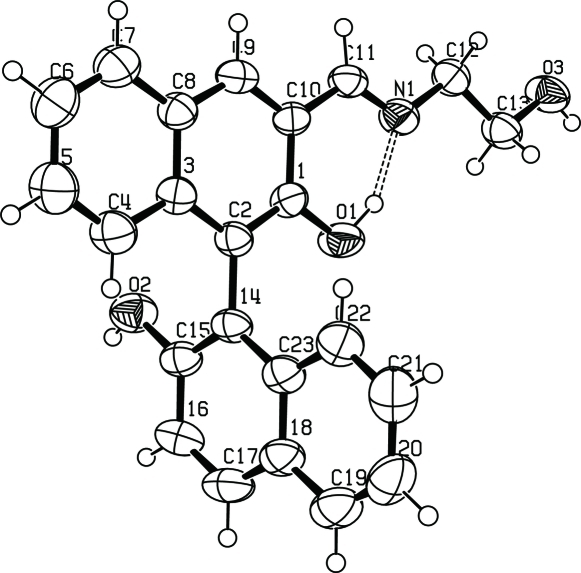
A perspective view of the title compound.

**Fig. 2.**
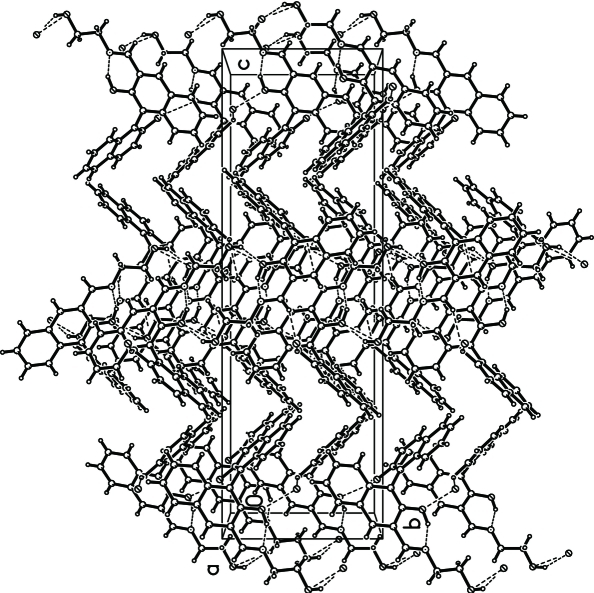
Intermolecular hydrogen bonding in the crystal structure of (I).

## supplementary crystallographic information

### Comment

BINOL and its derivatives have been largely used in asymmetric catalysis and chiral recognition (Pu, 1998). In this paper we present X-ray crystallographic analysis of the title compound (I), as the continuation of our previous studies.

As shown in Figure 1, an intramolecular O—H···N hydrogen bond between the hydroxy and the imino moieties forms a ring.

In the crystal, the molecules are connected by O—H···O hydrogen bonds ( Fig. 2).

### Experimental

The salen ligand,

3-((2-hydroxyethylimino)methyl)-1,1'-binaphthol was prepared by condensation of 3-carboxaldehyde-1,1'-binaphthol with 2-aminoethanol. Crystals suitable for X-ray analysis were obtained by slow evaporation of a ethanol /methylene chloride (1:5) solution of the compound.

### Refinement

All   H atoms except the one bonded to O1 (which was freely refined) were placed in calculated positions and refined in the riding-model approximation with O—H = 0.82Å and C—H = 0.93 or 0.97 Å) using a riding model with *U*_iso_(H) = 1.2 *U*_eq_(C,O).

### Crystal data

**Table:**

C_23_H_19_NO_3_	*D*_x_ = 1.291 Mg m^−^^3^
*M**_r_* = 357.39	Mo *K*α radiation, λ = 0.71073 Å
Orthorhombic, *P**b**c**a*	Cell parameters from 3454 reflections
*a* = 12.6184 (3) Å	θ = 2.7–22.4°
*b* = 9.7774 (2) Å	µ = 0.09 mm^−^^1^
*c* = 29.7991 (6) Å	*T* = 296 K
*V* = 3676.47 (14) Å^3^	Block, red
*Z* = 8	0.50 × 0.40 × 0.36 mm
*F*(000) = 1504	

### Data collection

**Table:**

Bruker SMART CCD area-detector diffractometer	4220 independent reflections
Radiation source: fine-focus sealed tube	1912 reflections with *I* > 2σ(*I*)
graphite	*R*_int_ = 0.089
φ and ω scans	θ_max_ = 27.6°, θ_min_ = 2.1°
Absorption correction: multi-scan (*SADABS*; Sheldrick, 2004)	*h* = −15→16
*T*_min_ = 0.661, *T*_max_ = 1.000	*k* = −12→12
24940 measured reflections	*l* = −38→38

### Refinement

**Table:**

Refinement on *F*^2^	Primary atom site location: structure-invariant direct methods
Least-squares matrix: full	Secondary atom site location: difference Fourier map
*R*[*F*^2^ > 2σ(*F*^2^)] = 0.055	Hydrogen site location: inferred from neighbouring sites
*wR*(*F*^2^) = 0.135	H atoms treated by a mixture of independent and constrained refinement
*S* = 1.00	*w* = 1/[σ^2^(*F*_o_^2^) + (0.0475*P*)^2^] where *P* = (*F*_o_^2^ + 2*F*_c_^2^)/3
4220 reflections	(Δ/σ)_max_ < 0.001
252 parameters	Δρ_max_ = 0.15 e Å^−^^3^
0 restraints	Δρ_min_ = −0.14 e Å^−^^3^

### Special details

**Table:**

Geometry. All e.s.d.'s (except the e.s.d. in the dihedral angle between two l.s. planes) are estimated using the full covariance matrix. The cell e.s.d.'s are taken into account individually in the estimation of e.s.d.'s in distances, angles and torsion angles; correlations between e.s.d.'s in cell parameters are only used when they are defined by crystal symmetry. An approximate (isotropic) treatment of cell e.s.d.'s is used for estimating e.s.d.'s involving l.s. planes.
Refinement. Refinement of *F*^2^ against ALL reflections. The weighted *R*-factor *wR* and goodness of fit *S* are based on *F*^2^, conventional *R*-factors *R* are based on *F*, with *F* set to zero for negative *F*^2^. The threshold expression of *F*^2^ > σ(*F*^2^) is used only for calculating *R*-factors(gt) *etc*. and is not relevant to the choice of reflections for refinement. *R*-factors based on *F*^2^ are statistically about twice as large as those based on *F*, and *R*- factors based on ALL data will be even larger.

### Fractional atomic coordinates and isotropic or equivalent isotropic displacement parameters (Å^2^)

**Table:**

	*x*	*y*	*z*	*U*_iso_*/*U*_eq_	
O1	0.50545 (11)	0.24608 (12)	0.43973 (4)	0.0661 (4)	
H1	0.5349 (18)	0.278 (2)	0.4675 (8)	0.109 (8)*	
O2	0.31307 (9)	−0.02597 (13)	0.38718 (4)	0.0615 (4)	
H2A	0.2483	−0.0321	0.3860	0.092*	
O3	0.60553 (9)	0.53599 (13)	0.59680 (4)	0.0647 (4)	
H3A	0.5790	0.6125	0.5963	0.097*	
N1	0.60154 (12)	0.25597 (15)	0.51551 (4)	0.0528 (4)	
C1	0.54441 (13)	0.11749 (16)	0.43397 (5)	0.0423 (5)	
C2	0.52157 (13)	0.04833 (17)	0.39500 (5)	0.0406 (4)	
C3	0.56310 (13)	−0.08531 (17)	0.38917 (5)	0.0424 (5)	
C4	0.54263 (15)	−0.16310 (18)	0.35028 (6)	0.0572 (6)	
H4A	0.5016	−0.1255	0.3275	0.069*	
C5	0.58156 (16)	−0.2919 (2)	0.34540 (7)	0.0696 (6)	
H5A	0.5678	−0.3406	0.3192	0.083*	
C6	0.64219 (16)	−0.3519 (2)	0.37943 (7)	0.0710 (6)	
H6A	0.6673	−0.4407	0.3761	0.085*	
C7	0.66425 (15)	−0.28066 (19)	0.41715 (7)	0.0617 (6)	
H7A	0.7059	−0.3207	0.4393	0.074*	
C8	0.62541 (13)	−0.14683 (17)	0.42360 (6)	0.0445 (5)	
C9	0.64473 (13)	−0.07085 (18)	0.46275 (6)	0.0478 (5)	
H9A	0.6848	−0.1104	0.4855	0.057*	
C10	0.60711 (12)	0.05847 (17)	0.46872 (5)	0.0392 (4)	
C11	0.62936 (13)	0.13284 (19)	0.51008 (5)	0.0452 (5)	
H11A	0.6693 (11)	0.0826 (15)	0.5322 (5)	0.044 (4)*	
C12	0.62678 (15)	0.32605 (17)	0.55768 (5)	0.0530 (5)	
H12A	0.7029	0.3270	0.5623	0.064*	
H12B	0.5943	0.2783	0.5827	0.064*	
C13	0.58577 (14)	0.46884 (18)	0.55525 (6)	0.0549 (5)	
H13A	0.6207	0.5177	0.5311	0.066*	
H13B	0.5102	0.4677	0.5492	0.066*	
C14	0.45087 (14)	0.11281 (17)	0.36073 (5)	0.0432 (5)	
C15	0.34642 (14)	0.07470 (18)	0.35878 (5)	0.0472 (5)	
C16	0.27531 (15)	0.1368 (2)	0.32883 (6)	0.0590 (6)	
H16A	0.2046	0.1099	0.3283	0.071*	
C17	0.31035 (17)	0.2365 (2)	0.30064 (6)	0.0650 (6)	
H17A	0.2626	0.2782	0.2812	0.078*	
C18	0.41683 (16)	0.27781 (19)	0.30024 (6)	0.0549 (5)	
C19	0.45588 (19)	0.3789 (2)	0.27067 (6)	0.0702 (6)	
H19A	0.4092	0.4217	0.2510	0.084*	
C20	0.5592 (2)	0.4152 (2)	0.27016 (6)	0.0761 (7)	
H20A	0.5832	0.4811	0.2501	0.091*	
C21	0.62985 (19)	0.3531 (2)	0.29999 (6)	0.0734 (7)	
H21A	0.7009	0.3783	0.2997	0.088*	
C22	0.59561 (16)	0.25552 (19)	0.32973 (6)	0.0593 (6)	
H22A	0.6437	0.2160	0.3495	0.071*	
C23	0.48854 (15)	0.21437 (18)	0.33073 (5)	0.0478 (5)	

### Atomic displacement parameters (Å^2^)

**Table:**

	*U*^11^	*U*^22^	*U*^33^	*U*^12^	*U*^13^	*U*^23^
O1	0.0975 (10)	0.0520 (8)	0.0489 (8)	0.0232 (7)	−0.0257 (8)	−0.0095 (6)
O2	0.0562 (8)	0.0747 (9)	0.0536 (8)	−0.0005 (7)	0.0008 (6)	0.0132 (7)
O3	0.0730 (8)	0.0630 (8)	0.0579 (8)	0.0175 (7)	−0.0196 (7)	−0.0187 (7)
N1	0.0659 (10)	0.0540 (10)	0.0385 (9)	0.0022 (8)	−0.0086 (8)	−0.0029 (7)
C1	0.0485 (10)	0.0401 (10)	0.0382 (10)	0.0048 (8)	0.0000 (9)	0.0015 (8)
C2	0.0447 (10)	0.0445 (10)	0.0325 (9)	0.0024 (8)	−0.0005 (8)	−0.0010 (8)
C3	0.0442 (10)	0.0481 (11)	0.0350 (10)	0.0015 (8)	0.0035 (8)	−0.0005 (8)
C4	0.0661 (13)	0.0612 (13)	0.0442 (11)	0.0094 (10)	0.0003 (10)	−0.0067 (10)
C5	0.0841 (15)	0.0675 (14)	0.0571 (13)	0.0148 (11)	0.0012 (12)	−0.0223 (11)
C6	0.0750 (14)	0.0562 (13)	0.0816 (15)	0.0182 (11)	−0.0013 (13)	−0.0155 (11)
C7	0.0581 (12)	0.0565 (12)	0.0704 (14)	0.0155 (10)	−0.0078 (11)	−0.0059 (11)
C8	0.0423 (10)	0.0461 (10)	0.0450 (10)	0.0051 (8)	−0.0011 (9)	−0.0022 (9)
C9	0.0427 (10)	0.0554 (11)	0.0454 (11)	0.0046 (9)	−0.0074 (9)	0.0066 (9)
C10	0.0424 (9)	0.0431 (10)	0.0322 (9)	0.0011 (8)	0.0007 (8)	0.0040 (8)
C11	0.0480 (10)	0.0522 (11)	0.0355 (10)	0.0006 (9)	−0.0055 (9)	0.0067 (9)
C12	0.0664 (12)	0.0519 (11)	0.0407 (11)	−0.0016 (10)	−0.0094 (10)	−0.0035 (9)
C13	0.0604 (12)	0.0573 (12)	0.0471 (11)	0.0057 (9)	−0.0105 (10)	−0.0088 (9)
C14	0.0518 (10)	0.0497 (11)	0.0282 (9)	0.0076 (8)	−0.0024 (8)	−0.0025 (8)
C15	0.0534 (11)	0.0523 (11)	0.0359 (10)	0.0084 (9)	0.0005 (9)	0.0014 (9)
C16	0.0555 (12)	0.0790 (14)	0.0426 (10)	0.0097 (10)	−0.0091 (10)	0.0000 (10)
C17	0.0763 (14)	0.0760 (14)	0.0425 (11)	0.0176 (11)	−0.0132 (11)	0.0057 (10)
C18	0.0778 (14)	0.0557 (12)	0.0312 (10)	0.0066 (10)	−0.0039 (10)	−0.0003 (9)
C19	0.1052 (17)	0.0666 (14)	0.0390 (12)	0.0089 (12)	−0.0046 (12)	0.0044 (10)
C20	0.1250 (19)	0.0609 (14)	0.0425 (12)	−0.0102 (13)	0.0083 (13)	0.0062 (10)
C21	0.0907 (16)	0.0754 (15)	0.0542 (13)	−0.0164 (12)	0.0142 (12)	−0.0060 (12)
C22	0.0715 (13)	0.0649 (13)	0.0414 (11)	−0.0009 (10)	0.0031 (11)	−0.0015 (10)
C23	0.0616 (12)	0.0521 (11)	0.0297 (10)	0.0056 (9)	0.0017 (9)	−0.0043 (9)

### Geometric parameters (Å, °)

**Table:**

O1—C1	1.3609 (19)	C10—C11	1.458 (2)
O1—H1	0.96 (2)	C11—H11A	0.964 (14)
O2—C15	1.3646 (19)	C12—C13	1.491 (2)
O2—H2A	0.8200	C12—H12A	0.9700
O3—C13	1.423 (2)	C12—H12B	0.9700
O3—H3A	0.8200	C13—H13A	0.9700
N1—C11	1.264 (2)	C13—H13B	0.9700
N1—C12	1.466 (2)	C14—C15	1.371 (2)
C1—C2	1.374 (2)	C14—C23	1.418 (2)
C1—C10	1.425 (2)	C15—C16	1.404 (2)
C2—C3	1.418 (2)	C16—C17	1.361 (3)
C2—C14	1.495 (2)	C16—H16A	0.9300
C3—C4	1.410 (2)	C17—C18	1.403 (3)
C3—C8	1.426 (2)	C17—H17A	0.9300
C4—C5	1.360 (3)	C18—C19	1.413 (3)
C4—H4A	0.9300	C18—C23	1.425 (2)
C5—C6	1.399 (3)	C19—C20	1.352 (3)
C5—H5A	0.9300	C19—H19A	0.9300
C6—C7	1.351 (3)	C20—C21	1.398 (3)
C6—H6A	0.9300	C20—H20A	0.9300
C7—C8	1.410 (2)	C21—C22	1.372 (3)
C7—H7A	0.9300	C21—H21A	0.9300
C8—C9	1.404 (2)	C22—C23	1.410 (3)
C9—C10	1.362 (2)	C22—H22A	0.9300
C9—H9A	0.9300		
			
C1—O1—H1	105.5 (13)	N1—C12—H12B	109.9
C15—O2—H2A	109.5	C13—C12—H12B	109.9
C13—O3—H3A	109.5	H12A—C12—H12B	108.3
C11—N1—C12	119.62 (14)	O3—C13—C12	109.21 (14)
O1—C1—C2	119.04 (15)	O3—C13—H13A	109.8
O1—C1—C10	118.87 (14)	C12—C13—H13A	109.8
C2—C1—C10	122.08 (15)	O3—C13—H13B	109.8
C1—C2—C3	118.63 (15)	C12—C13—H13B	109.8
C1—C2—C14	119.65 (15)	H13A—C13—H13B	108.3
C3—C2—C14	121.68 (14)	C15—C14—C23	119.07 (15)
C4—C3—C2	121.99 (15)	C15—C14—C2	119.20 (15)
C4—C3—C8	117.70 (15)	C23—C14—C2	121.71 (15)
C2—C3—C8	120.29 (15)	O2—C15—C14	117.79 (15)
C5—C4—C3	121.41 (17)	O2—C15—C16	120.60 (16)
C5—C4—H4A	119.3	C14—C15—C16	121.60 (17)
C3—C4—H4A	119.3	C17—C16—C15	119.64 (18)
C4—C5—C6	120.56 (18)	C17—C16—H16A	120.2
C4—C5—H5A	119.7	C15—C16—H16A	120.2
C6—C5—H5A	119.7	C16—C17—C18	121.51 (18)
C7—C6—C5	119.96 (19)	C16—C17—H17A	119.2
C7—C6—H6A	120.0	C18—C17—H17A	119.2
C5—C6—H6A	120.0	C17—C18—C19	122.73 (18)
C6—C7—C8	121.33 (18)	C17—C18—C23	118.52 (17)
C6—C7—H7A	119.3	C19—C18—C23	118.75 (19)
C8—C7—H7A	119.3	C20—C19—C18	121.8 (2)
C9—C8—C7	122.93 (16)	C20—C19—H19A	119.1
C9—C8—C3	118.05 (15)	C18—C19—H19A	119.1
C7—C8—C3	119.02 (16)	C19—C20—C21	119.6 (2)
C10—C9—C8	122.62 (16)	C19—C20—H20A	120.2
C10—C9—H9A	118.7	C21—C20—H20A	120.2
C8—C9—H9A	118.7	C22—C21—C20	120.8 (2)
C9—C10—C1	118.31 (15)	C22—C21—H21A	119.6
C9—C10—C11	120.41 (15)	C20—C21—H21A	119.6
C1—C10—C11	121.27 (15)	C21—C22—C23	120.92 (19)
N1—C11—C10	121.97 (16)	C21—C22—H22A	119.5
N1—C11—H11A	122.9 (9)	C23—C22—H22A	119.5
C10—C11—H11A	115.1 (9)	C22—C23—C14	122.30 (16)
N1—C12—C13	108.70 (14)	C22—C23—C18	118.10 (17)
N1—C12—H12A	109.9	C14—C23—C18	119.61 (17)
C13—C12—H12A	109.9		
			
O1—C1—C2—C3	179.60 (15)	C11—N1—C12—C13	179.82 (16)
C10—C1—C2—C3	−0.8 (2)	N1—C12—C13—O3	177.37 (14)
O1—C1—C2—C14	−2.7 (2)	C1—C2—C14—C15	−99.84 (19)
C10—C1—C2—C14	176.94 (15)	C3—C2—C14—C15	77.8 (2)
C1—C2—C3—C4	179.57 (16)	C1—C2—C14—C23	78.9 (2)
C14—C2—C3—C4	1.9 (2)	C3—C2—C14—C23	−103.43 (19)
C1—C2—C3—C8	1.2 (2)	C23—C14—C15—O2	178.20 (14)
C14—C2—C3—C8	−176.48 (15)	C2—C14—C15—O2	−3.0 (2)
C2—C3—C4—C5	−178.97 (17)	C23—C14—C15—C16	−2.0 (3)
C8—C3—C4—C5	−0.6 (3)	C2—C14—C15—C16	176.74 (15)
C3—C4—C5—C6	0.9 (3)	O2—C15—C16—C17	−179.66 (16)
C4—C5—C6—C7	−1.3 (3)	C14—C15—C16—C17	0.6 (3)
C5—C6—C7—C8	1.4 (3)	C15—C16—C17—C18	1.0 (3)
C6—C7—C8—C9	178.55 (18)	C16—C17—C18—C19	178.59 (18)
C6—C7—C8—C3	−1.0 (3)	C16—C17—C18—C23	−1.0 (3)
C4—C3—C8—C9	−179.00 (15)	C17—C18—C19—C20	−178.56 (19)
C2—C3—C8—C9	−0.6 (2)	C23—C18—C19—C20	1.0 (3)
C4—C3—C8—C7	0.6 (2)	C18—C19—C20—C21	−1.0 (3)
C2—C3—C8—C7	179.02 (16)	C19—C20—C21—C22	0.2 (3)
C7—C8—C9—C10	179.89 (17)	C20—C21—C22—C23	0.6 (3)
C3—C8—C9—C10	−0.5 (2)	C21—C22—C23—C14	179.33 (17)
C8—C9—C10—C1	1.0 (2)	C21—C22—C23—C18	−0.5 (3)
C8—C9—C10—C11	179.91 (15)	C15—C14—C23—C22	−177.84 (16)
O1—C1—C10—C9	179.33 (15)	C2—C14—C23—C22	3.4 (3)
C2—C1—C10—C9	−0.3 (2)	C15—C14—C23—C18	2.0 (2)
O1—C1—C10—C11	0.4 (2)	C2—C14—C23—C18	−176.76 (15)
C2—C1—C10—C11	−179.21 (15)	C17—C18—C23—C22	179.31 (17)
C12—N1—C11—C10	−179.51 (15)	C19—C18—C23—C22	−0.3 (3)
C9—C10—C11—N1	175.33 (16)	C17—C18—C23—C14	−0.5 (3)
C1—C10—C11—N1	−5.7 (3)	C19—C18—C23—C14	179.91 (16)

### Hydrogen-bond geometry (Å, °)

**Table:**

*D*—H···*A*	*D*—H	H···*A*	*D*···*A*	*D*—H···*A*
O2—H2A···O3^i^	0.82	1.87	2.6638 (17)	161
O3—H3A···O1^ii^	0.82	2.05	2.7724 (17)	147
O1—H1···N1	0.96 (2)	1.67 (2)	2.5649 (18)	153 (2)

Symmetry codes: (i) *x*−1/2, −*y*+1/2, −*z*+1; (ii) −*x*+1, −*y*+1, −*z*+1.
